# MolADI: A Web Server for Automatic Analysis of Protein–Small Molecule Dynamic Interactions

**DOI:** 10.3390/molecules26154625

**Published:** 2021-07-30

**Authors:** Bing Bai, Rongfeng Zou, H. C. Stephen Chan, Hongchun Li, Shuguang Yuan

**Affiliations:** 1 Shenzhen Institutes of Advanced Technology, Chinese Academy of Sciences, 1068 Xueyuan Avenue, Shenzhen 518055, China; bing.bai@siat.ac.cn (B.B.); rf.zou@siat.ac.cn (R.Z.); xc.chen@siat.ac.cn (H.C.S.C.); 2 Shenzhen Institute of Advanced Technology, University of Chinese Academy of Sciences, 1068 Xueyuan Avenue, Shenzhen 518055, China; 3 AlphaMol Science Ltd., 1068 Xueyuan Avenue, Shenzhen 518055, China

**Keywords:** protein–ligand interaction, trajectory analysis, WebGL, web server

## Abstract

Protein–ligand interaction analysis is important for drug discovery and rational protein design. The existing online tools adopt only a single conformation of the complex structure for calculating and displaying the interactions, whereas both protein residues and ligand molecules are flexible to some extent. The interactions evolved with time in the trajectories are of greater interest. MolADI is a user-friendly online tool which analyzes the protein–ligand interactions in detail for either a single structure or a trajectory. Interactions can be viewed easily with both 2D graphs and 3D representations. MolADI is available as a web application.

## 1. Introduction

The Protein Data Bank (PDB) [1] has nearly 180,000 deposited protein structures, of which over 75% form complexes with small molecules. The binding of a ligand to its partner protein requires a specific arrangement of attractive forces, usually non-covalent contacts, between the two molecules. With such a wealth of data, we can gain insights into how ligands interact with their protein targets and modify the interactions by replacing functional groups in ligands [2,3]. To date, there are only a few open source tools for analyzing and visualizing protein–ligand interactions, especially for the analysis of protein–small molecule trajectories. Here, we build an efficient and dynamic website for the analysis of protein–small molecule interactions, and the visualization of these information in various forms, including radar chart, heat map, and 3D dynamic display.

MolADI is an extension to other websites (such as PLIP [4], SwissDock [5], GalaxySite [6], or ProBiS [7]) with its own unique advantages: using a structure and a trajectory as inputs, users can pinpoint whether certain specific interactions exist in a particular frame and keep track their variations along the dynamic trajectory. At the same time, according to the results of the 2D heat map, users can obtain the overall interaction information from all trajectories which facilitates the ligand chemical space optimization.

MolADI also provides the atomic information that users can easily obtain the specific information of certain interactions, including the name of the atom, the position in the data file, and the detailed information of the interaction. The website also provides user manuals and tutorials, so that users can familiarize with MolADI easily.

## 2. Results

The MolADI website provides a simple and clear interface for protein–small molecule dynamic visualization. The original intention of the entire website is to enable users to analyze and obtain results more easily. Users only need to upload the data files they want to analyze and can obtain the results quickly. After testing, the analysis time is about 30 s, which is more efficient than manual analysis. The result pages also provide a wealth of visual results, including 2D and 3D results. The information of each interaction between protein and small molecule can be clearly viewed, such as the location where the interaction occurs, the distance and angle of the interaction, and the interaction changes in the entire trajectory, etc. At the same time, MolADI’s multiple filter functions also allow users to locate different interactions of small molecules more easily to further analyze their specific information in the binding site of the receptor, such as how the distance and angle change in the dynamic trajectory.

### Example

The MolADI homepage provides the analysis result of a test file. The user can enter the analysis result of the test file by clicking the “Example” button on the homepage. The test file contains one small molecules and multi-trajectories, which include three types of interactions (hydrophobic, hydrogen bond, and pi-stack) with 12 residues. Users can make use of the filtering function of the functional area, which enables location and view of specific information for each interaction. Users can also get relevant test data in the GitHub (https://github.com/whiteice-c/moladi (accessed on 28 June 2021)).

**Figure 1:**
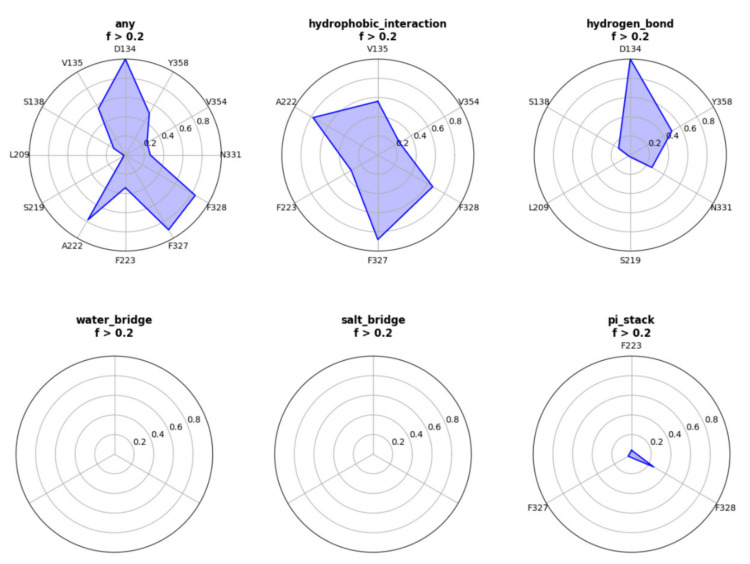

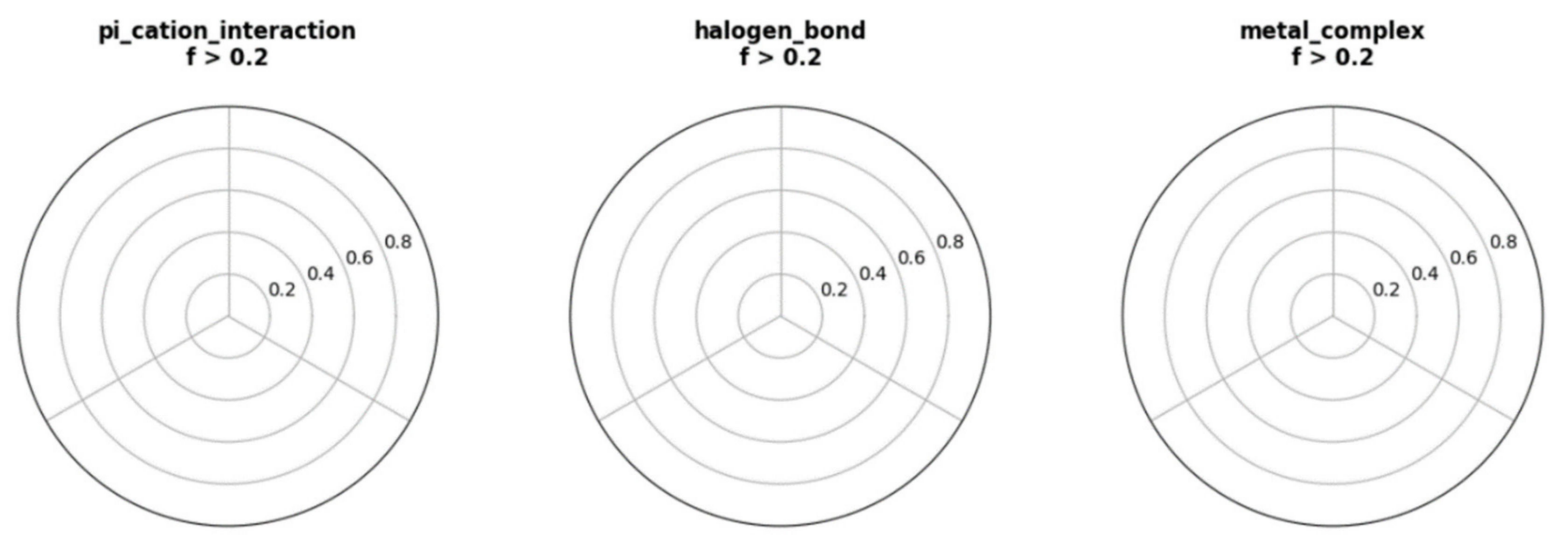
Interactive radar distribution map results of a ligand (UNK) bind to A2AR.

We used this webserver to analysis a trajectory related to a ligand (UNK) bind to A2AR. The residues that have contact frequency with ligand higher than 20% of all the frames are shown Figure 1. The hydrophobic interaction contributes mostly to the protein–ligand interactions. Residues—including V135, A222, F223, F327, F328, and V354—have hydrophobic interaction with ligand higher than 20% of all the frames. We also see that D134 forms hydrogen bond interactions with the ligand. Other interactions, such as water-bridge, salt-bridge, pi-stack, contribute slightly or have no contributions to the protein–ligand interactions.

**Figure 2:**
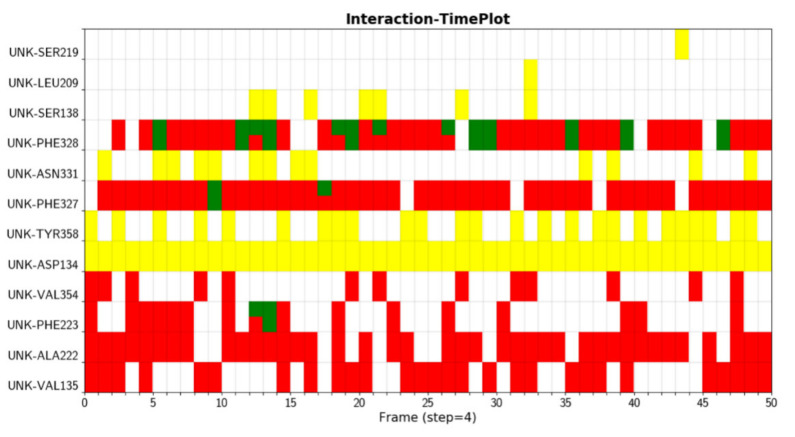
Interactions between protein (A2AR) and ligand (UNK) are displayed with respect to simulation time.

We were able to see the changes of interactions with respect to simulation time in Figure 2, which is useful to assess the stabilities of interactions. Here different colors indicate different types of interaction. The hydrogen bond formed between D134 and ligand (yellow) is very stable, as it is formed during the whole simulation. The hydrophobic interaction between F328 and ligand does not exist in the crystal structure, but can be formed in the simulation, which could be due to the rearrangements of ligands and residues.

**Figure 3:**
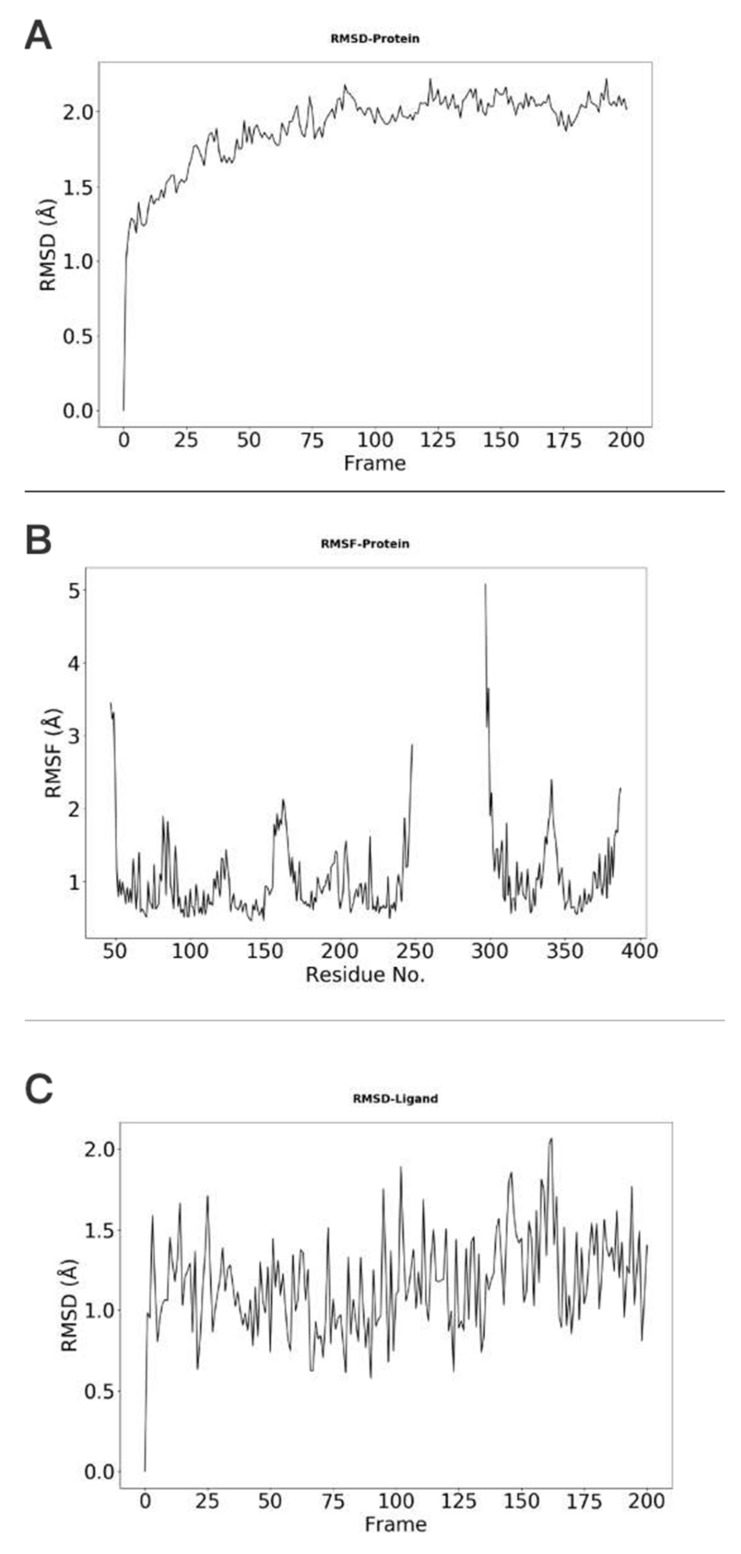
Conformational changes and residue flexibilities of the protein and conformational changes of the ligand. (**A**) The protein Cα-RMSDs results indicate the conformational changes of the A2AR. (**B**) The protein Cα-RMSFs results show the flexibility or fluctuation of residues of the protein. (**C**) The ligand RMSDs indicate the conformational changes of the UNK.

The stability of protein, which is indicated by the RMSD (root-mean-square deviation) changes with respect to simulation time, is shown in Figure 3A. In this example, the protein RMSD values are all below 3.0 Å, suggesting that the conformation of the protein is stable during the simulation. The RSMF (root-mean-square fluctuation) plot can also be obtained with our webserver. RMSF can be used to describe the flexibility of a residue in the simulation. The larger the RMSF value is, the more flexible the residue is. As shown in Figure 3B, residues with RMSF value higher that 1.5 Å correspond to those in the flexible loop region. The RMSD of ligand is shown in Figure 3C, showing that the ligand does not change its orientations significantly during the simulation.

To be able to visualize the 3D structures of the simulated system, we integrated the NGL viewer in our webserver. All the types of interactions can be shown with dash lines with different colors. It is also possible to view these interactions at different frames.

## 3. Discussion

MolADI is a website that provides interactive visualization services of protein–small molecule interactions. Its simple design and fast analysis speed greatly reduce the operation difficulty and provide good interactive experience for users. It allows users to obtain results in a short time after uploading files, and provides rich visualization results to reflect the results from multiple aspects, including the radar chart which reflects the frequency of the interaction, the interaction information distribution map of the entire trajectory, the RMSD and RMSF reflecting the stability of the protein structure and the 3D dynamic visualization, and also a downloading function of the results. MolADI is a prototype for further development. For the analysis of trajectory files, MolADI selects 50 frames through equidistant sampling instead of using all frames to analyze the interaction information. The main reason here is that visualizing more trajectories will cause the front-end to respond much more slowly, leading to unsatisfactory experience of observing 3D dynamic changes. Therefore, we chose fewer frames to analyze by the server for faster response. In addition, if there is excessive interaction information in the user’s data file (although this situation is rare), the visualization effects, such as radar charts, will become less clear.

For a future version of MolADI, we plan to optimize and improve the website services—such as accepting more file types, providing complete trajectory visualization, and the download for standalone version—so that users can analyze and observe the results through local tools. In addition, we will publish script files to enable professional users to run large data files on their own machines.

## 4. Materials and Methods

Workflow and implementation: MolADI aims at helping users analyze protein–ligand interactions more conveniently and visualize the results better than currently available tools. MolADI provides both 2D and 3D display with control panels. Python scripts were used to generate the radar chart and heat map for the 2D display, while the NGL (JavaScript) library was used to show protein structures and protein–ligand interactions for the 3D display [8,9]. Although several companies, such as the Chemical Computing Group (MOE), Accelerys and CLC bio, provide similar software, tools, web pages and databases [10,11,12,13,14,15,16,17,18,19], many of these are commercial software and can only be used for visualization purposes. Other available tools only provide limited choices of interaction types, and require a large amount of input files or preventing processing of specified structures. Therefore, MolADI aims to solve these problems and provide better visualizations.

The workflow of MolADI is implemented with the Django web framework and MariaDB database. First, the structure and trajectory files uploaded by user will be analyzed using the PLIP method and customized Python scripts [4]. Then, the results will be displayed on the results page using NGL viewer with WebGL technology.

Input: A structure file in PDB format and a trajectory file in any of xtc, gro, and dcd formats should be selected as input. After jumping to the analysis interface, the front-end page will display some current analysis progress information in real time. The average time for analyzing 50 frames is about 30 s.

Output: The result contains three pages, namely the overview page, the 3D result display page, and the 2D result display page.

The overview of the result page is divided into two areas. The left side provides 2D interaction information map displayed in the form of a radar chart. The right side provides the 3D interaction information. The function column contains the corresponding residue information, the atom pair in interaction and their distance. The area in the page can be zoomed and moved to adjust the viewing angle.

The initial result contains all the interaction information related to small molecule in the data file. In the function column on the right, users can select the interaction information of interest to visualize. The functionalities are listed below:

- Customizing the display information about the types of interactions of interest. There are nine selection boxes in this functional area in total, including the eight specific interaction types, as well as an option to display all of the interactions or not. Users can click on the selection box of the corresponding types to decide whether to display them. The color corresponding to the interaction type is also marked in this area, so that users can understand the results better.
- Customizing small molecules for selections. This function is used to select which interaction information of small molecule will be displayed. All interactions are displayed by default. All the small molecules are listed here. By selecting the interaction of a specific small molecule of interest to display, the contents of two regions (2D region and 3D region) will correspondingly be changed to show the information of the selected small molecule.
- Option to display the interaction information only. This function is for users to easily observe the interaction information. By default, the structure diagram will be displayed. However, if the interaction information is too complicated, the structure information may mask the interaction information. This function can be used to display only the interaction information to better observe the corresponding interaction information.
- Center. This function is used to locate the 3D result to the center of the page to facilitate the observation of the result.

The above functional areas can be used freely and flexibly to meet the needs for different observation requirements. Finally, the result download function is provided in the upper right corner of the overview page, and the result can be saved as a .pdf file to local machine. It mainly includes 2D result information.

**Figure 4:**
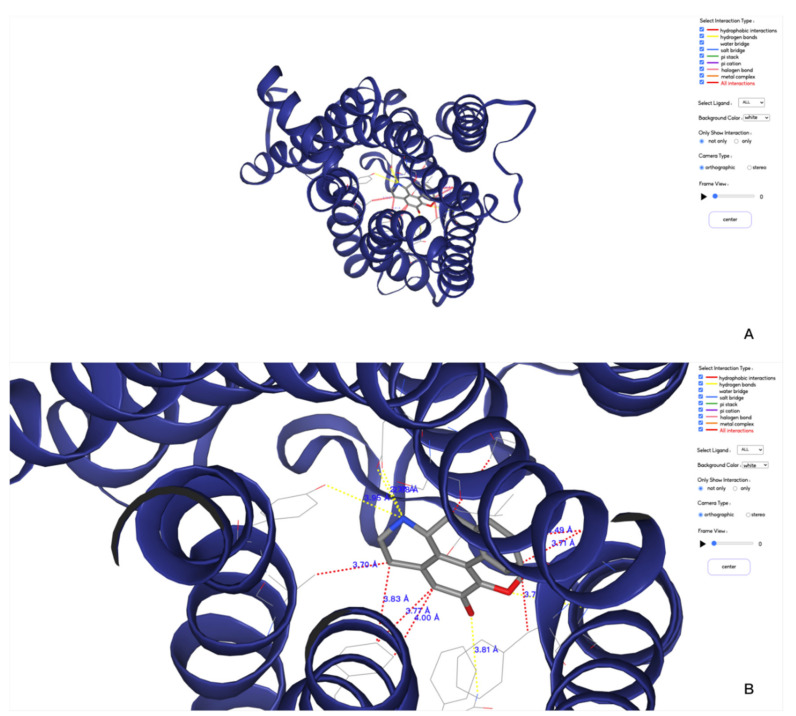
3D visualizations of protein–ligand interactions between protein A2AR and ligand UNK. (**A**,**B**) are a default view and a zoomed in view of the 3D displaying, respectively.

The function of the 3D display page that indicated in Figure 4 provides a larger area for users to visualize the results. Compared to the overview page, a dynamic result view has been added to this page: by clicking the play button in the functional area, the 3D result will display the trajectory and the interaction information from each frame sequentially in an infinite loop. The movie can be paused to locate the frame of interest. More conveniently, by scrolling the axis on the right side of the button, the user can directly locate a frame for visualization. In addition, two small functions have been added. One is to change the background color of the 3D view. There are three color options, so that users can choose the appropriate background color when saving the picture. The other is to select the view mode, the default is orthographic mode, the user can also select the stereo mode, and then wear 3D glasses to observe the stereo view effect.

**Figure 5:**
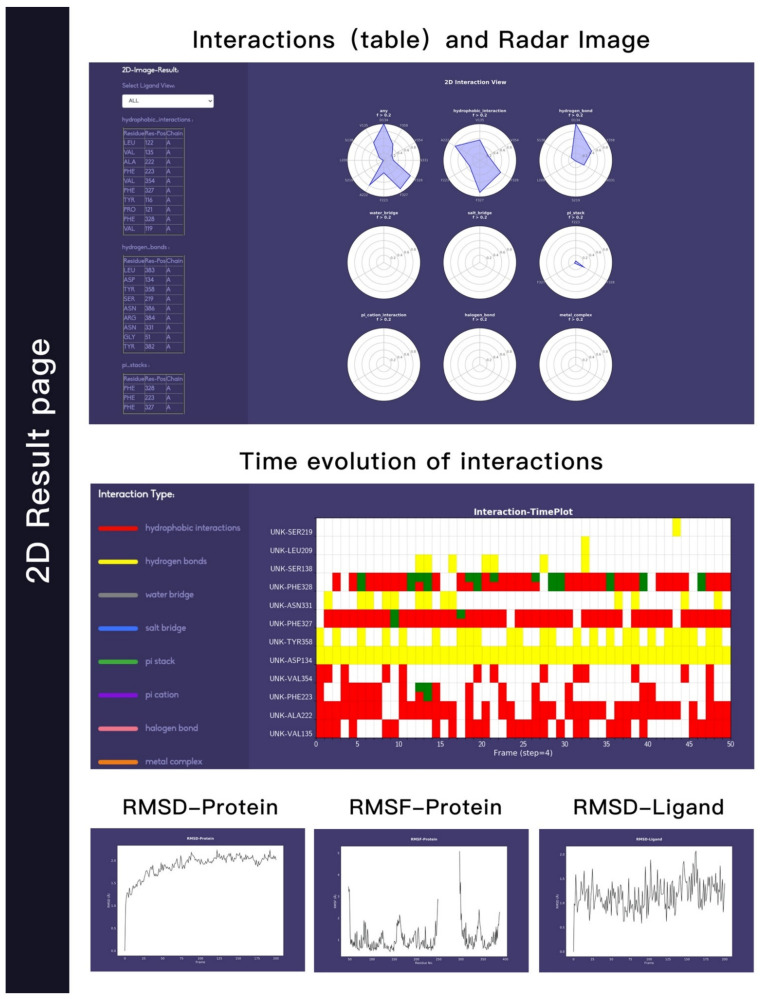
2D visualizations of protein–ligand interactions and flexibility of both protein A2AR and ligand UNK. The first row mainly displays the interaction in the form of a table and a radar chart. The second row is in the form of a heat map, and the third row is the RMSD and RMSF information.

Figure 5 shows the 2D result page, which mainly reflects the interaction information through 2D graphs. In the left side, all interactions are listed in table form. On the right, the first row is the radar chart on the overview page. Note that the list and the radar chart are in one-to-one correspondence. The corresponding interaction can also be selected and displayed through the small molecule selection function (top left). The second row on the right is the heat map of interaction, which indicates the dynamic interaction information in the 2D form. It shows the interaction variations along the input trajectory. The *x*-axis shows the frame serial number. In this Figure, a total of 50 frames are selected from the trajectory file uploaded by the user at an intermediate interval. The *y*-axis shows the specific interaction information, including the interaction type, residues and/or the small molecules involved and the small molecule ID. From the heat map, users can clearly see the dynamic changes of a specific interaction in the entire trajectory. The bottom row reflects the stability of the protein structure in the trajectory. The backbone–backbone RMSD, backbone–backbone RMSF, and backbone–ligand RMSD are shown.

There are three main steps in computational analysis by MolADI: data preprocessing, interactive information calculation, and information visualization.

MolADI first preprocesses the input files uploaded by users. For the trajectory file the number of frames is calculated from the original file, from which 50 equidistant frames are combined into a single .pdb file as a new trajectory file. Redundant information will be removed to prevent errors in the subsequent calculation of frame information. The original data file is used when calculating RMSD and RMSF. Next, the calculation of interaction information will be carried out by PLIP, which undergoes four steps to detect and report relevant interactions: structure preparation, functional characterization, rule-based matching and filtering of interactions. PLIP uses OpenBabel [20] for internal representation of molecules and most chemoinformatics calculations, and rule-based matching and interactive filtering. Visualization is achieved after getting the interaction information file. After the server obtains the result file of interactive information, it will perform simple processing and then render the information for visualization.

2D visualization: The radar chart records the frequency of each interaction and the number of frames in the file to calculate the probability of each interaction, and then highlight the probability of each interaction. For the heat map, the server will traverse the result file, record each interaction and its corresponding serial number, and then draw the heat map. Each row in the heat map represents an interaction shown with its serial number set. It reflects the change of interaction in the dynamic trajectory. For multi-frame RMSD and RMSF, the server uses the corresponding formulas for root mean square error and root mean square fluctuation.

3D visualization: 3D visualization is implemented based on NGL open-source code. The NGL Viewer is a web application for the visualization of macromolecular structures. it supports common structural file-formats (e.g., PDB, mmCIF) and a variety of molecular representations (e.g., ‘cartoon’, ‘spacefill’, ‘licorice’). Moreover, the viewer can be embedded in other web sites to provide specialized visualizations of the entries in structural databases or results of structure-related calculations [9,10]. Based on NGL, MolADI customizes its own personalized 3D visualization functions, which mainly include interaction filters, small molecule filters, and dynamic analysis functions.

## Data Availability

Users can get relevant test date in the (https://github.com/whiteice-c/moladi (accessed on 28 June 2021)).

## Sample Availability

MolADI is freely accessible at http://cadd.siat.ac.cn/moladi/ (accessed on 28 June 2021).
